# The role of agriculture in achieving Universal Health Coverage in Africa

**DOI:** 10.7189/jogh.12.03053

**Published:** 2022-07-16

**Authors:** Shadrack Osei Frimpong, Sten H Vermund

**Affiliations:** 1 Yale School of Public Health, Yale University, New Haven, Connecticut, USA; 2 Department of Public Health and Primary Care, University of Cambridge, Cambridge, UK

The World Health Organization strives to assist nations towards “the attainment by all peoples of the highest possible level of health” within the context of the Sustainable Development Goals (SDGs) [1]. The Universal Health Coverage (UHC) policy is the first joint United Nations resolution calling for all countries worldwide to provide affordable, equitable, and quality health care for all their citizens. In the quest for UHC, global health policies and programs have highlighted the need for control of infectious diseases such as HIV/AIDS, tuberculosis, malaria, and COVID-19, as well as non-communicable diseases (NCDs) and maternal and child health. As rural residents in low- and middle-income countries (LMICs), especially those in the farming communities, typically have lower access to quality education and health care, farm families are often disadvantaged. However, development programs usually miss the inextricable links between agriculture, health, and education [2]. They rarely share the same forums of policy or discourse. Given UHC's importance in addressing acute and chronic diseases, including pandemic threats, agriculture, education, and health can be leveraged more strategically towards achieving UHC.

In this viewpoint, we discuss how agriculture can be harnessed to achieve UHC through revenue generation from cash crop production, purchase of user fees with improved farmer income levels, prevention of diseases, and the control of obesity and its associated NCDs. Since case studies have often been used to examine social innovations due to their heterogeneous nature, we further employ a case study of Cocoa360s model to show how agriculture can be leveraged to finance health and educational access, two major social determinants of health.

## AGRICULTURE AND UNIVERSAL HEALTH COVERAGE: SOME EVIDENCE FROM THE LITERATURE

### Agricultural revenues as sources of health financing

Many successful UHC initiatives in LMICs rely on significant increases in taxation and public spending, requiring income generation. While external donor funding can catalyse LMICs to finance their UHC efforts, domestic health resources must be increased to ensure that predictable and sustainable funding is available. Ghana is one such nation that can improve its domestic fundraising towards UHC through cocoa revenues. As the world's second-leading exporter of cocoa, Ghana earns about US $2 billion in yearly export revenue from the crop [3]. Still, its once highly touted National Health Insurance Scheme (NHIS) faces significant financing challenges that could lead to its discontinuation. Only second to mining, Ghana’s cocoa sector contributes 3% to the national gross domestic product (GDP), covering about 20%-30% of the country’s total export receipts [3]. Government investments in fertilizers, pesticides, and farm machinery for cocoa farmers would lead to higher crop yields. Such gains could generate new tax revenues that can finance the NHIS. While cash crop production can affect local production of healthy diets and increase market prices of foodstuffs, it can also raise the income levels of farmers who depend on them for their daily access to food [4]. With enhanced incomes, these farmers can cover their health insurance premiums and other out-of-pocket health care expenses. An inability to cover such costs keeps financial risk protection out of the reach of many of Africa's rural farmers and fisher folks.

### Agricultural productivity and income can help improve health outcomes

Over 800 million people around the globe still experience undernutrition, either from deficiencies of macronutrients like proteins or micronutrients such as vitamins [5]. About half the adults worldwide are overweight or obese, leading to rising NCDs such as diabetes and hypertension [5]. In many LMICs, childhood malnutrition in stunting, severe wasting, and intrauterine growth restriction accounts for about 2.2 million deaths and a 21% of the disability-adjusted life years of children aged under five years [6]. High mortality and disease burdens resulting from these nutrition-related issues make an undeniable case for the need to leverage agriculture to address them and integrate nutrition into UHC policies.

Agricultural productivity enables many households to produce, purchase, and consume better and sometimes cheaper food. Recently, biofortification efforts to breed and distribute crops rich in micronutrients such as vitamin A and iron have improved vitamin and mineral consumption in Africa and Asia [7]. Agriculture's impact extends beyond disease prevention; economically, it saves the most lives with the highest return on investment, with every dollar invested yielding, on average, US $16 on the return [7].

### Agricultural innovations can prevent diseases

Failure to integrate agriculture's links to health as a crucial disease-prevention tool will undermine UHC efforts. For instance, proper agricultural water management has reduced malaria-related deaths. Shifting irrigation approaches to a yearly wet crop/dry crop rotation cycle can significantly minimise vector breeding [2]. For about 70% of the world's rural poor, endemic zoonosis is a significant risk factor for reduced income generation from livestock and disease infections. Such diseases include faecal-oral bacteria (eg, *Campylobacter spp*., *Escherichia coli*., *Leptospira interrogans*), zoonotic tuberculosis (TB), vesicular stomatitis, and parasites (eg, African trypanosomiasis, giardiasis) [8]. Irrigation can be associated with schistosomiasis because of expanded snail breeding zones, and sluices from dams may spawn onchocerciasis due to oxygenated water favourable for blackfly breeding. Also, better food storage can reduce toxic metabolites such as aflatoxins that contribute to hepatocellular carcinoma. Additionally, experimental farm and animal husbandry practices can reduce zoonotic disease risk, improve farm yields, and enhance ecologic sustainability (**Table 1**).

**Table 1:** Suboptimal practices in agriculture and their associated diseases

Suboptimal agricultural practice	Associated diseases and conditions
Over cultivation, failure of crop rotation, wrong crop cultivation	Protein-calorie malnutrition, kwashiorkor
Residual water in agricultural and household borrow pits or containers	Malaria, filariasis, dengue, zika virus; other arthropod-borne parasitic and viral diseases
Pesticide exposure	Respiratory tract, eye, and skin irritation, cancer, asthma, possibly Parkinson disease
Presence of aflatoxins	Foodborne cancer exposures, food spoilage
Irrigation and sluices	Schistosomiasis, onchocerciasis
Antibiotic use for animal growth	Antibiotic-resistant bacterial infections
Poor drainage, tilling, and grazing practices, excessive deforestation	Soil erosion and loss of topsoil, flooding, river/lake pollution desertification
Urbanization, global warming	Loss of farmland; Disrupted crops cycles*

*Reduced crop yields in tropical environments; a paradox of greater yields in cold climates.

### Agriculture and social determinants of health: Case study of Cocoa360

**Figure 1:**
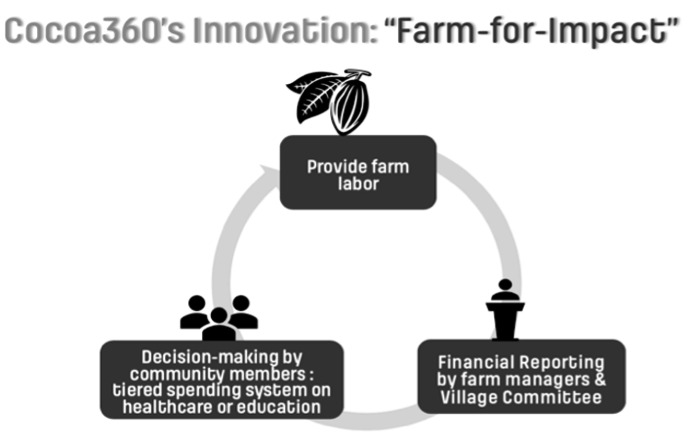
Cocoa360s Farm-For-Impact Model.

One of the authors (SOF) established a global health nonprofit organisation, Cocoa360, in his home village of Tarkwa Breman in western Ghana. Despite high national cocoa export revenue, cocoa farmers are trapped in a cycle of poverty and grapple with health care and educational access. In response, Cocoa360 was established to improve education and medical services access. By the end of 2021, Cocoa360s 60-acre campus comprised the Tarkwa Breman Girls' School, the Tarkwa Breman Community Clinic, and the Tarkwa Breman Community Farm, collectively serving eight rural communities. Cocoa360 has pioneered a “farm-for-impact” (FFI) model: community members work on community-run cocoa farms in exchange for tuition-free education for their children and access to subsidised health care services. Cocoa360s history, impact, and the FFI model are detailed elsewhere [9]. A schematic of the FFI model is demonstrated in **Figure 1** [9].

**Figure:**
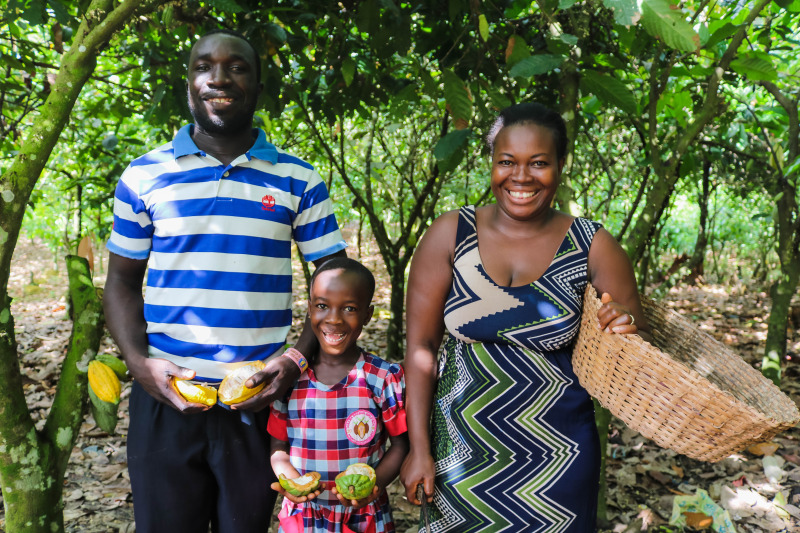
Photo: Parents of a student at Cocoa360s Tarkwa Breman Girls' School, working on Cocoa360s farm. Source: from Cocoa360, used with permission.

### Cocoa360 uses agriculture to fund education, a social determinant of health

Education and family health are correlated positively. Education benefits are pronounced in children – educated young boys and girls are more knowledgeable about infectious diseases and are more likely to adopt preventive behaviours and attitudes that lower infection risks. A field study in four Ghanaian villages with a high HIV prevalence suggested that cognitive and decision-making abilities resulted in health-protective behaviours rather than specific HIV/AIDS knowledge. Such skills increase with access to formal schooling [10]. Health-related benefits of education become critical during public health emergencies, as with the COVID-19 pandemic, where adherence to preventive measures such as physical distancing is required.

Significant discrepancies in educational attainment due to poverty and gender preferences persist between the sexes in LMICS such as Ghana. Consequently, the Ghanaian government implemented the Free Compulsory Universal Basic Education (FCUBE) policy to provide tuition-free primary education to all young girls and boys in public schools [11]. While the FCUBE has increased primary school enrolment rates, it appears that eliminating tuition costs is not sufficient to eliminate gender disparities in educational access. Many young girls do not benefit from this scheme, likely because other school-related expenses such as uniforms, textbooks, and transportation remain a significant financial burden for poor households [11].

These challenges guided Cocoa360s decision and collaboration with community members in establishing a girls-only school in rural Ghana. Through the FFI model, Cocoa360 has worked with community members to apply cocoa farm revenues towards non-tuition expenses such as books and uniforms [9]. Preliminary findings from Cocoa360 show that the school attendance rate at the Tarkwa Breman Girls' School (TBGS) is 98% compared to the national rural school attendance rate of 70% [9]. The organisation plans to partner with the Ghanaian government to strengthen other rural public schools in any of the over 1300 cocoa-growing communities across the country. In this partnership, the government would continue to pay teachers' salaries in its schools. Simultaneously, Cocoa360 would then leverage its community engagement lessons to fund the non-tuition expenses that the government does not cover.

### How Cocoa360 leverages agriculture to pre-finance health care user fees

While remarkable strides towards UHC have been made, health financing challenges persist. In Ghana, this situation is acute in rural communities where farming families may not access health care due to financial barriers such as user fees [12]. Reduction in these fees was a primary motive for the government instituting the NHIS. The scheme's mission was bold: to achieve equity for all Ghanaians through financial risk pooling. However, its benefits have been limited to only those who have registered for and are members of the NHIS. This coverage dilemma has, unfortunately, further widened the health care inequity gap. The poor, who are based mainly in rural areas, have been most affected, as they are unable to afford the required annual premium membership fee, which varies regionally, from GH¢10 (US$2.09) to GH¢20 (US$4.17) [12].

In LMICs, farmers are at an increased risk of poor health conditions due to their exposure to farming risks. The many rural Ghanaians not covered under NHIS must make out-of-pocket payments such as user fees, resulting in their reduced use of health care services. Studies show that removing user fees increases facility-based deliveries, improved primary care access, and overall improved health outcomes for underserved communities. However, no community-led pre-financing mechanism for user fees has been implemented in Ghana to cover such costs, excluding funding or support from external donors [13].

Cocoa360 seeks to apply its “farm-for-impact” model to reduce user fees in response to this challenge. The organisation's success in implementing the FFI model to subsidize educational expenses serves as a guiding strategy to abate health care user fees. These may be the cost of diagnostic and maternity services that persons who are not registered under the NHIS cannot access. Although Cocoa360 might be perceived as a transient competitor of the public health system, it seeks to be a “strengthener” of NHIS eventually. By partnering with Ghana's Ministry of Health, Cocoa360 seeks to work with communities to leverage the FFI model towards eliminating user fees to ensure higher health care services utilization and improved health outcomes.

## CONCLUSION

**Figure 2:**
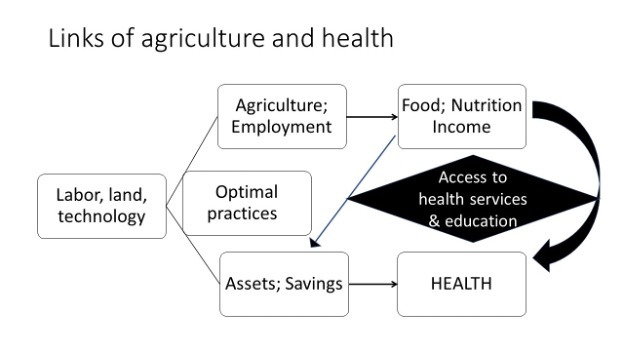
Relationship of agriculture with health.

When the WHO celebrates its 75th anniversary on April 7, 2023, the global community will review its UHC attainment initiatives and their role in combatting acute pandemics such as COVID-19 and endemic challenges such as NCDs. Achieving UHC by 2030 will take more than a narrow focus on disease prevention and treatment. Affiliated sectors such as agriculture and education will have to be leveraged to address the persistent challenges of health financing, prevention of infectious and non-communicable diseases, financial risk protection, as well as public health and medical care quality (**Figure 2**). Cocoa360 is an informative integrated model for financing health care and education through agricultural (communal farming) activities in rural areas towards achieving UHC.

## References

[1] Brault MA Mwinga K Kipp AM Kennedy SB Maimbolwa M Moyo P Measuring child survival for the Millennium Development Goals in Africa: what have we learned and what more is needed to evaluate the Sustainable Development Goals? Glob Health Action. 2020;13:1732668. 10.1080/16549716.2020.1732668 32114967 PMC7067162

[2] Dubé L Pingali P Webb P Paths of convergence for agriculture, health, and wealth. Proc Natl Acad Sci U S A. 2012;109:12294-301. 10.1073/pnas.0912951109 22826252 PMC3412024

[3] Ghana Statistical Service (GSS) Statistics for Development and Progress Annual Gross Domestic Product 2015. Available: https://www.statsghana.gov.gh/gssmain/fileUpload/National%20Accounts/2015_Annual_GDP_September_2015_Edition.pdf. Accessed: 20 June 2022.

[4] Achterbosch TJ, van Berkum S, Meijerink GW, Asbreuk H, Oudendag DA. Cash crops and food security: contributions to income, livelihood risk and agricultural innovation. Available from: https://research.wur.nl/en/publications/cash-crops-and-food-security-contributions-to-income-livelihood-r. Accessed: 19 May 2022.

[5] Humphries DL, Scott ME, Vermund SH. Pathways linking nutritional status and infectious disease: causal and conceptual frameworks. In Nutrition and infectious diseases, nutrition and healthCham, Switzerland: Humana; 2021.

[6] Black RE Allen LH Bhutta ZA Caulfield LE de Onis M Ezzati M Maternal and child undernutrition: global and regional exposures and health consequences. Lancet. 2008;371:243-60. 10.1016/S0140-6736(07)61690-0 18207566

[7] 2018 Global Nutrition Report - Global Nutrition Report. Global Nutrition Report. 2019. Available: https://globalnutritionreport.org/reports/global-nutrition-report-2018/. Accessed: 15 September 2021.

[8] Bardosh KL Scoones JC Grace D Kalema-Zikusoka G Jones KE de Balogh K Engaging research with policy and action: what are the challenges of responding to zoonotic disease in Africa? Philos Trans R Soc Lond B Biol Sci. 2017;372:20160172. 10.1098/rstb.2016.0172 28584180 PMC5468697

[9] Frimpong S, Russel AR, Handy F. Re-imagining community development: the Cocoa360 model, In: Research Handbook on Community Development. Northampton MA: Edward Elgar Publishing; 2020.

[10] Peters E Baker DP Dieckmann NF Leon J Collins J Explaining the effect of education on health: a field study in Ghana. Psychol Sci. 2010;21:1369-76. 10.1177/0956797610381506 20739672

[11] Nudzor HP Taking education for all goals in sub-Saharan Africa to task: What’s the story so far and what is needed now? Manage Educ. 2015;29:105-11. 10.1177/0892020615584105

[12] Kotoh AM Van der Geest S Why are the poor less covered in Ghana’s national health insurance? A critical analysis of policy and practice. Int J Equity Health. 2016;15:34. 10.1186/s12939-016-0320-1 26911139 PMC4766646

[13] Kanchebe Derbile E van der Geest S Repackaging exemptions under National Health Insurance in Ghana: how can access to care for the poor be improved? Health Policy Plan. 2013;28:586-95. 10.1093/heapol/czs098 23065542

